# High resolution lectin microarray system for the ATL cell-typing and the evaluation of anti-cancer efficacy of medications

**DOI:** 10.1186/1742-4690-11-S1-O68

**Published:** 2014-01-07

**Authors:** Hidekatsu Iha, Emi Ikebe, Takashi Matsumoto, Akira Nishizono, Masao Ogata, Yuetsu Tanaka, Mitsuo Hori, Eisaburo Sueoka, Jun-ichi Fujisawa, Kazuhiro Morishita

**Affiliations:** 1 Department of Infectious Diseases, Oita University, Japan; 2 Department of Pathology, Faculty of Medicine, Oita University, Japan; 3 Department of Immunology, Graduate School of Medicine, University of Ryukyus, Japan; 4 Department of Hematology, Ibaragi Pref. Central Hospital, Japan; 5 Department of Hematology, Saga University, Faculty of Medicine, Japan; 6 Department of Microbiology, Kansai Medical University, Japan; 7 Department of Medical Sciences, Faculty of Medicine, University of Miyazaki, Japan

## 

Regardless of species, all the living organisms have poly-saccharides called glycans in their cellular surfaces or even inside the cells. Glycans interact with sugar-binding proteins, lectin, and affects on the numerous biological activities such as development, immune response, organogenesis or even cancer development. Although there are many reports on the expressional disorders of lectins or glycans in cancer cells, most of them have described only single or few gene product's aberrations.

Here we describe the application of a novel lectin microarray system (LecMS), which consists of 45 lectins with different binding preferences and provides the multiple glycan-lectin binding intensity information, on various leukemic cell lines.

As we carry the endemic areas of Adult T-cell Leukemia (ATL), which is caused by an infection of retrovirus (HTLV-1), our interest was focused on how this system characterize the ATL or non-ATL cells by their lectin-glycan binding intensity (LGI) values.

From the results of LecMS profiling on thirteen different ATL or non-ATL tumor cells, we found LGI values which are commonly induced in all tumor cells and values induced specifically in ATL cells, respectively. Furthermore, we found these LGI values change with anti-cancer drug administration and the changing patterns varies with cell lines. High-throughput, sensitive and relatively simple nature of this system could be utilized as the novel profiling tool for the prognosis of ATL.

